# Parkinson’s Disease: A Narrative of the Evolving Understanding of the Role of α-Synuclein in Screening

**DOI:** 10.3390/cimb46110757

**Published:** 2024-11-10

**Authors:** Alan D. Kaye, Kassady A. Perkinson, Noah J. Spillers, Alexis J. Vega, Caylin J. Roberts, Evan M. Downs, Melissa M. Sheth, David W. McGregor, Shahab Ahmadzadeh, Jibin Mathew, Sahar Shekoohi

**Affiliations:** 1 Department of Anesthesiology, Louisiana State University Health Sciences Center, 1501 Kings Highway, Shreveport, LA 71103, USA; 2 Department of Pharmacology, Toxicology and Neurosciences, Louisiana State University Health Sciences Center, 1501 Kings Highway, Shreveport, LA 71103, USA; 3 Edward Via College of Osteopathic Medicine, Monroe, LA 71203, USA; kperkinson@ulm.vcom.edu; 4 School of Medicine, Louisiana State University Health Sciences Center, Shreveport, LA 71103, USAcjr001@lsuhs.edu (C.J.R.);; 5 Medical City Weatherford, 713 E Anderson St, Weatherford, TX 76086, USA

**Keywords:** α-synuclein, Parkinson’s disease screening, seeding, amplification assay

## Abstract

The present investigation aims to examine the role of α-synuclein seed amplification assays for screening Parkinson’s disease. Parkinson’s disease (PD) is a debilitating neurodegenerative disorder caused by the loss of dopaminergic neurons in the midbrain, leading to symptoms such as tremors, bradykinesia, postural instability, dementia, and depression. It is classified as an α-synucleinopathy related to the role of α-synuclein aggregates in neuron degeneration. Diagnosis relies on clinical assessment without premortem diagnostic tests or imaging, often resulting in delayed detection and impaired symptom management. In this regard, our study explores a screening technique using an amplification assay to measure α-synuclein levels in cerebrospinal fluid, which could potentially identify early pathological changes and improve diagnostic accuracy and patient outcomes. While preliminary results are promising, further studies are needed to evaluate this approach’s accuracy and clinical feasibility. A review of numerous trials demonstrates that α-synuclein seeding amplification assays (SAA) are a highly reliable, sensitive, and specific diagnostic tool for PD. This assay offers a promising opportunity to improve early diagnosis and quantify severity, especially for asymptomatic individuals or those with a family history of PD, allowing for earlier intervention and more effective disease management. In summary, the emerging body of evidence supporting α-synuclein as a biomarker should allow patients with PD to be detected and treated sooner, enhancing patients’ quality of life and potentially changing the disease trajectory.

## 1. Introduction

Parkinson’s disease (PD) is a neurodegenerative disorder that progressively worsens over time [1]. There are four principal motor symptoms seen in PD that aid in making this diagnosis. These symptoms include resting tremors, bradykinesia (slow/hesitant movements), postural instability, and hypertonia [2]. The fourth symptom, hypertonia, is commonly described as “freezing”; although this symptom is a cardinal feature of PD, it is not always observed [2]. Beyond the four symptoms listed above, additional symptoms, including non-motor features, are also seen [2]. The non-motor symptoms of PD include autonomic dysfunctions, sleep disorders, cognitive and neurobehavioral disorders, and sensory abnormalities [2]. These symptoms are much less telling of PD as they can also be seen in other neurodegenerative disorders [2]. The overlap of symptoms between PD and other neurodegenerative disorders suggests the need to explore alternative diagnostic methods, potentially focusing on specific changes seen within the brains of those suffering from PD.

The motor and non-motor changes seen in PD are suspected to be related to loss of dopaminergic neurons and accumulation of α-synuclein (referred to as Lewy bodies on histological staining) [3]. Dopaminergic neurons located in the substantia nigra pars compacta of the midbrain are responsible for motor control. Loss of neurons and accumulation of α-synuclein in the substantia nigra serves as an explanation for the symptoms commonly seen in those with PD [3]. Looking less at the loss of dopaminergic neurons and more at the accumulation of α-synuclein in patients with PD could be a breakthrough in diagnosing PD.

PD has a prevalence of 572 per 100,000 in North America, with an estimated total economic burden of USD 51.9 billion in the US alone in 2017 [4,5]. The prevalence coupled with the cost of care and treatment of this debilitating disease makes it an enticing target for researchers to develop more effective diagnostic and therapeutic tools to combat the disease.

It has been previously demonstrated that seed amplification assays (SAA) developed for the detection of α-synuclein in the cerebral spinal fluid (CSF) have a sensitivity of 100% in detecting α-synuclein in patients with synucleinopathies, including PD, and are moderately successful in differentiating between those different synucleinopathies, i.e., differentiating PD from other movement disorders, e.g., multiple system atrophy (MSA) [6]. This review aims to take a closer look into the utilization of α-synuclein seed amplification assays in successfully screening for PD. In the present investigation, we evaluate the role that α-synuclein has in Parkinson’s disease, the techniques involved in the utilization of α-synuclein seeding amplifications assays, and analyze some of the current clinical studies that utilize these methods in diagnosing PD.

## 2. Materials and Methods

This was a narrative review. The sources for this review are as follows: searching on PubMed, Google Scholar, Medline, and ScienceDirect using the keywords Parkinson’s disease, α-synuclein, seed amplification assay, and neurodegenerative disorders. Other keywords include dopamine, dopaminergic neurons, and Alzheimer’s disease. Sources were accessed between December 2023 and July 2024, and the literature search had no limitations for publication. Additional studies on seed amplification assays were not included.

## 3. Parkinson’s Disease Diagnosis and Screening

Most cases of PD are sporadic, although some genetic risk factors have been identified. PD has an average onset in the fifth decade of life, with incidence increasing with age, and is characterized by both cognitive decline and progressive motor dysfunction leading to rigidity, resting tremor, postural instability, dementia, and depression in the later stages [7,8].

Risk factors for Parkinson’s disease consist of both environmental and genetic factors and include exposure to pesticides, rural living, a family history of PD, and a family history of tremors [9,10]. Toxic gain of function mutations in the gene LRRK2 coding for dardarin, loss of protective function mutations in the genes PRKN, PINK1, and DJ-1, as well as substitution mutations affecting the SNCA gene coding for α-synuclein, have all been implicated in the pathogenesis of PD [11,12,13,14,15].

A diagnosis of PD is made in the clinical setting, and symptoms typically have a gradual onset. The earliest signs of PD are known as the premotor phase and are not specific to the disease. These symptoms include rapid eye movement, sleep disorders, loss of smell, mood disorders, constipation, depression, and excessive daytime sleepiness [16]. This phase of the disease often has an insidious onset lasting for years before the onset of motor symptoms. Classic motor symptoms such as stiffness, instability, and tremor typically begin later in the disease process once roughly half of neurons in the caudal substantia nigra have been lost [17,18].

To date, no diagnostic tests are widely available to aid clinicians in detecting PD, leaving the diagnosis largely up to physician judgment. This often leads to delays in the recognition of symptoms and delays in eventual diagnosis and treatment [17,19,20,21]. Diagnostic tissue and fluid biomarker assays focusing primarily on quantification of α-synuclein aggregation in blood and cerebrospinal fluid are currently in development, but none have progressed to clinical relevance [20,22,23].

Minimally invasive diagnostic tests for the evaluation of suspected PD cases could play a major role in the future treatment and progression of PD. Confirmatory tests are not currently available for living patients with a diagnosis of PD, necessitating the progression of motor and non-motor symptoms to validate preliminary clinical suspicion [21]. Early identification and confirmatory tests will be required to utilize the best advanced disease-modifying drugs and therapies. Initiation of these future treatment options at the earliest possible point in the disease process will offer patients the most therapeutic benefit, and could potentially alter the course and progression of the disease [20].

## 4. α-Synuclein Role in Parkinson’s + Seeding Amplification Assay

### 4.1. Pathophysiology

PD is considered a multisystemic α-synucleinopathy involving the loss of dopaminergic neurons in the substantia nigra pars compacta, a structure located in the midbrain, as well as Lewy body deposition that can involve the CNS and other tissues. The pathophysiology of the destruction of these dopaminergic neurons has been linked to several pathways, including mitochondrial dysfunction, aggregation and misfolding of the neuroprotein α-synuclein, and oxidative stress due to the high metabolic demand of these neurons [24,25]. Intraneural inclusion bodies composed of aggregates of α-synuclein, today known as Lewy bodies, were first described by Dr. Friedrich Heinrich Lewy in 1812 and have since become pathognomonic of the disease [26].

α-synuclein’s native structure is typically unfolded. However, it demonstrates significant conformational plasticity and is susceptible to undergoing misfolding that is highly dependent upon its environment [27]. Once misfolded, it is prone to forming morphological variants, including amyloid fibrils, protofibrillar oligomeric intermediates, and amorphous aggregates that all serve as the genesis for the formation of Lewy bodies [27,28].

As these aggregates are formed, they serve as a nucleus for the rapid aggregation of wild-type α-synuclein, exacerbating the deposition of both mutated and wild-type forms of the protein [29]. As these insoluble protein complexes aggregate, they become cytotoxic by interfering with normal mitochondrial dynamics and causing nuclear degradation, leading to neuronal dysfunction and, eventually, cell death [30].

### 4.2. α-Synuclein as a Biomarker for Parkinson’s Disease

PD patients are usually diagnosed when motor symptoms prompt the patient to seek medical treatment. This is usually many years after the onset of neuropathic changes due to α-synuclein misfolding and aggregation in the central nervous system, and the diagnosis cannot be confirmed until autopsy [31]. Currently, no diagnostic biomarkers are available or approved for diagnosing Parkinson’s disease. The ability to detect misfolded α-synuclein in PD patients would allow us to diagnose the disease significantly earlier and facilitate clinical trials targeting the early stages of neurodegeneration [31]. Earlier medical treatment for PD would have the potential to enhance therapeutic intervention for patients by the ability to prevent permanent brain damage suffered by current PD patients [32]. An ideal biomarker would be readily quantifiable inaccessible samples, such as blood or cerebrospinal fluid (CSF). It should be able to detect a significant increase or decrease in the relevant disease without being influenced by other factors. A biomarker that reflects the underlying pathophysiology of PD would likely meet the above criteria for an ideal diagnostic biomarker. As described above, multiple studies reveal α-synuclein as having a role in the neuropathology of PD, including its misfolding and aggregation in the central and peripheral nervous systems. Many studies target α-synuclein in the peripheral nervous system (PNS), as it has been hypothesized that neurodegeneration begins in the PNS and spreads centrally, thereby aiming for pre-motor diagnosis [31].

Biochemical markers of α-synuclein in the saliva, blood, and CSF are being actively investigated as potential diagnostic tools, as they are much easier to access than tissue. α-synuclein in the blood is a potential diagnostic tool because α-synuclein might be transported to the blood from the central nervous system [31]. Rodent studies have shown that labeled α-synuclein injected into the brain can be detected in plasma, and α-synuclein levels were higher in PD subjects than healthy controls. Still, total plasma α-synuclein levels were not significantly different [33]. CSF is a relatively accessible biofluid. It is commonly utilized for neurologic diagnostic assays: albuminocytologic dissociation for Guillain–Barre syndrome, oligoclonal bands for multiple sclerosis, and autoantibodies for autoimmune encephalitis.

At present, CSF α-synuclein is the most extensively studied biomarker for PD. Inspiration for utilizing CSF α-synuclein came from CSF biomarkers developed for Alzheimer’s disease, including amyloid-β1–42 and total tau and phosphorylated tau. These CSF biomarkers are now included in research diagnostic criteria for both prodromal and dementia stages of Alzheimer’s disease (AD) due to reduced levels of amyloid and elevated levels of tau on threonine 181 in CSF showing 85–95% sensitivity and specificity for AD at the stage of dementia or earlier mild cognitive impairment stage [34,35]. Unfortunately, the same diagnostic success in biomarker development has not been achieved in PD [34].

### 4.3. Seeding Amplification Assays

Seed amplification assays (SAAs) are similar to PCR for genetic material but used for proteins. SAAs are commonly used for the diagnosis of Creutzfeldt–Jakob disease, a prion subacute encephalopathy. The pathophysiology of prion disorders, such as Creutzfeldt–Jakob and Kuru, involves the abnormal proteins PrPSc eliciting a conformational change that favors polymerization. These polymerized proteins collect in the brain and cause permanent brain damage. When SAAs are utilized for α-synuclein biospecimens, they are put into a reaction chamber with excess recombinant α-synuclein (rec-αSyn). Any α-synuclein seeds in the specimen will elicit a conformational change in rec-αSyn and promote fibrillization.

Mechanical disruption of emerging aggregates causes fragments to seed further polymerization and amplify the biomarker to detectable quantities. The generated fibrils of α-synuclein contain β-sheets, enabling the amyloid-specific dye Thioflavin (ThT) detection [36]. Misfolded α-synuclein has recently been successfully detected in the CSF, olfactory mucosa, skin, saliva, and submandibular gland biopsies of patients with PD and other synucleinopathies. Studies have shown these assays have the ability to differentiate PD and dementia with Lewy bodies (DLB) and multiple system atrophy (MSA) from non-synucleinopathy parkinsonism [32]. The kinetic analysis of SAA fluorescence profiles allows for detecting synucleinopathy-dependent α-synuclein fibril conformations, which reflect differences among synucleinopathies such as Lewy body diseases (DLB and PD) and MSA. This data has demonstrated α-synuclein detected by SAAs as the most promising biomarker for synucleinopathies in general but not specifically for PD [31].

A 2023 cross-sectional analysis by Siderowf et al. revealed that α-synuclein SAA can differentiate PD patients with high sensitivity and specificity. SAA provides information about molecular heterogeneity based on whether they had PD due to α-synuclein pathology or not, and it can identify patients in the latency asymptomatic period of the disease [37]. The study examined at-risk and prodromal individuals through different cohorts. The study examined heterogeneity by utilizing multiple cohorts of CSF samples from 1100 participants in the PPMI (Parkinson’s progression marker initiative). These cohorts included CSF samples from 1100 individuals in the PPMI with the genetic form of Parkinson’s disease who are currently diagnosed, carriers of PD genes, prodromal patients with clinical features of PD but negative dopamine imaging, at-risk patients, and healthy controls. The at-risk patients were patients with REM sleep behavior disorder and hyposmia with positive dopamine transmitter imaging. In “The Neurology podcast”, Dr. Siderowf describes the findings of the study as follows: “Among typical PD cohorts, SAA was positive 98% of the time, positive carrier status SAA was positive 2/3 of the time and sporadic PD with normosmia was positive 2/3 of the time. LARK2 cohorts (a genetic form of PD) showed fewer positive results, indicating that SAA detects synuclein pathology rather than non-specific pathology, such as dopamine depletion. Patients with no Lewy pathology tend to be LARK2 carriers and normosmic. About 2/3 LARK2 PD have Lewy pathology, and 1/3 do not, which matches with the study results showing 2/3 positive SAA and 1/3 negative”. In other words, these results propose that α-synuclein SAA is a reliable test for typical Lewy body synucleinopathy. Based on the findings of the study, α-synuclein SAA could potentially play a crucial role in identifying pathologically defined subgroups of people with Parkinson’s disease and establishing biomarker-defined at-risk cohorts [38].

## 5. Clinical Studies of α-Synuclein Amplification Assay

A new method of diagnosing Parkinson’s disease (PD) is needed. Several studies have explored the efficacy of the α-synuclein seed amplification assay (SAA) as a potential tool in diagnosing Parkinson’s disease. A few of these studies are discussed below.

### 5.1. Pilot: University of Edinburgh—2016

The first study exploring this topic was conducted in Scotland in 2016. The investigators set out to develop an assay that detects aggregations of α-synuclein in the CSF and brain tissue from patients diagnosed with dementia with Lewy bodies (DLB) or PD. They utilized real-time quaking-induced conversion (RT-QuIC) with a reaction buffer that included human recombinant α-synuclein. The assay was found to have 92% sensitivity in detecting DLB (meaning 92% of the samples known to be DLB-positive gave a positive result in the assay) and 95% sensitivity in detecting PD. It was 100% specific when compared to CSF or brain tissue from patients with Alzheimer’s disease (AD) or control subjects, meaning none of the AD or control samples gave a positive result. The study also found that the assay showed a positive response for three subjects with REM sleep behavior disorder (RBD) who had an increased risk for developing a synucleinopathy, which suggests that the assay may be able to provide a diagnosis prior to the development of motor symptoms. Corticobasal degeneration (CBD) and progressive supranuclear palsy (PSP) are two movement disorders that are commonly mistaken for PD. These disorders, however, are caused by a dysfunctional tau protein rather than α-synuclein. The investigators tested CSF from patients with these two diseases. They found that they gave a negative result in the assay, which shows the assay may be used to differentiate among the diseases when clinical symptoms are non-specific. This study was the first to demonstrate the possible clinical utility of a diagnostic test for PD that may be used before the patient displays symptoms and provoked further investigation of the assay in many studies since then [39].

### 5.2. University of Texas—2020

Another trial set out to determine if the assay (here termed “protein misfolding cyclic amplification” or PMCA) can differentiate between the CSF of a patient with Parkinson’s disease and one with multiple system atrophy (MSA), another synucleinopathy. In the PMCA assay, the fluorescence of thioflavin T (ThT) indicates the aggregation of α-synuclein. Data from this trial showed that the maximum ThT fluorescence was significantly greater in CSF samples from patients with Parkinson’s disease than those with MSA. Aggregation profiles for each sample were also studied. A total of 4 of the 75 MSA samples had an aggregation profile typical of PD (84.6% specificity), and of the 94 PD samples, 3 had a profile compatible with MSA (93.6% specificity). This team also amplified brain homogenates from cadavers that had been diagnosed with PD or MSA and found that the amplified α-synuclein aggregates showed profiles typical of their respective diagnosis, also found in CSF. This suggests that the α-synuclein aggregates in the CSF represent those in the brain and confirms the utility of living patients’ CSF in the assay. This study also provides evidence that the assay can differentiate PD from MSA based on aggregation profile [40].

### 5.3. Amprion—2021

Another study used CSF samples from the Parkinson’s Progression Markers Initiative (PPMI) cohort, a longitudinal study that collects biological samples, including CSF, and patient data to create a comprehensive data set and biosample library for PD research purposes [41]. Patients in the PPMI cohort were subjected to a DaTscan, a form of brain imaging used to determine the degeneration of dopaminergic neurons, at different intervals following enrollment into the study. Three groups of CSF were studied: patients who had been diagnosed with PD with evidence of dopaminergic deficit on DaTscan, patients clinically diagnosed with PD but without evidence of dopaminergic deficit (classified as SWEDD), and healthy controls. They used samples obtained from the patients at enrollment into the PPMI and their three-year follow-up visit and ran the samples through an α-synuclein PMCA. The assay was found to have 96.2% sensitivity and 96.7% specificity for PD versus control at baseline and 96.4% sensitivity and 93.8% specificity three years later. Interestingly, the assay seemed to have picked up on α-synuclein present in CSF before dopaminergic deficits could be seen on DaTscan. Of the 20 patients who were labeled as SWEDD at enrollment, 4 were found to be positive for α-synuclein by the assay. Two of these patients were found to have dopaminergic degeneration on DaTscan consistent with PD at four years post-enrollment. These results show that the α-synuclein PMCA assay is very accurate in determining PD from individuals with normal DaTscans and may be able to detect changes before they may be seen on a scan, enabling an earlier diagnosis and, therefore, treatment [42].

### 5.4. Amprion, AbbVie, Caughey Comparison

One study was conducted to compare the α-synuclein SAA methods used by three different laboratories (AbbVie, Amprion, and Caughey) and determine the reproducibility of the assay across providers and labs. Each lab used CSF from the PPMI database collected at enrollment and the three-year follow-up. The groups of patients studied were patients diagnosed with PD, SWEDD, and healthy controls.

### 5.5. Qatar Biomedical Research Institute—2022

One clinical trial explored the efficacy of combining α-synuclein SAA with ELISA to provide an accurate, quantitative test result that correlates with disease severity. While SAA provides a positive-or-negative response based on whether pathologic α-synuclein is present, ELISA can link to α-synuclein oligomers to determine their total concentration. They first used postmortem brain tissue from cases with a confirmed diagnosis of Parkinson’s disease, dementia with Lewy bodies (DLB) (another synucleinopathy), or Alzheimer’s disease (AD) (a non-synucleinopathy.) The data from this cohort showed that the SAA-ELISA multiplex consistently identified PD and DLB from AD and controls. The data also showed that adding ELISA provides quantitative discrimination between the groups and results up to 50 h faster than SAA alone.

They then obtained CSF from patients diagnosed with Parkinson’s disease and age-matched healthy controls. After 20 h in the SAA, samples were analyzed with ELISA assays for α-synuclein oligomers or total α-synuclein. The levels of α-synuclein oligomers were significantly higher in PD patients compared to control, while the total α-synuclein levels did not differ significantly. The levels of α-synuclein oligomers also strongly correlated with motor function, as classified by both the Unified Parkinson Disease Rating Scale (UPDRS) and Hoehn and Yahr (H&Y) score. This trial provides strong evidence that the addition of ELISA to the SAA protocol may be used to make an earlier diagnosis and determine the severity of the disease at a quantitative level [43].

A summary of the studies above can be found in Table 1. Additional recent studies exploring the assay are summarized in Table 2.

## 6. Discussion

Early detection of Parkinson’s disease and effective treatment are critical for clinical care. However, despite advancements in research, current diagnostic methods often fail to identify PD until after substantial neurodegeneration has already occurred, complicating treatment efforts and limiting patients’ options for maintaining their quality of life. The progressive nature of PD means that early diagnosis could drastically improve outcomes by allowing treatment to start earlier in the disease course. Early intervention could help preserve motor function and cognitive abilities, significantly improving patients’ ability to maintain independence. Moreover, for individuals with a family history of PD, the availability of screening tools could provide an invaluable opportunity to assess their risk and take proactive measures long before symptoms appear, potentially delaying disease onset or reducing its impact. The search for an ideal biomarker for PD continues to be a major focus of research. A biomarker that is easily measurable, found in accessible samples such as blood or cerebrospinal fluid (CSF), and accurately reflects disease progression without being influenced by other variables would revolutionize PD diagnosis and treatment.

One promising candidate is α-synuclein (α-synuclein), a protein involved in the pathophysiology of PD. Researchers have been exploring the detection of α-synuclein in various bodily fluids, including saliva, blood, and, most notably, CSF. The relative ease of accessing CSF and its established role in other neurological diagnostic tests make it a key area of focus for PD biomarkers. α-synuclein seed amplification assays (SAAs) have emerged as one of the most promising diagnostic approaches for detecting synucleinopathies. Although α-synuclein has not yet been conclusively shown to be specific for PD, data from numerous studies suggest that the SAA method offers high diagnostic accuracy. It can identify patients in the asymptomatic phase of the disease and differentiate PD caused by α-synuclein pathology from other neurodegenerative disorders, such as multiple system atrophy (MSA). One of the most significant findings from these studies is the potential for the α-synuclein PMCA assay to detect PD earlier than traditional imaging techniques. In addition to diagnostic accuracy, reproducibility is crucial for any screening or diagnostic test. A study examining the consistency of results across three independent laboratories demonstrated high sensitivity and specificity for α-synuclein SAA, reinforcing its reliability as a diagnostic tool. This consistency is essential for screening programs, where results must be reproducible regardless of where or how the test is conducted. Another promising area of research involves the combination of SAA and ELISA, which has been shown to enhance diagnostic accuracy and even quantify the severity of PD, providing clinicians with a more comprehensive understanding of the disease’s progression in individual patients. Further studies have also shown a strong correlation between α-synuclein levels and the progression of PD, highlighting its potential not only as a diagnostic marker but also as a tool for monitoring disease progression and evaluating the effectiveness of treatments. This could lead to more personalized approaches to managing PD, where treatment plans are tailored to each patient’s specific needs based on their disease’s severity and progression.

## 7. Conclusions

In summary, the body of research surrounding the α-synuclein seeding amplification assay (SAA) underscores its potential as a reliable, sensitive, and specific diagnostic tool for PD. This assay could significantly enhance early diagnosis, particularly for asymptomatic individuals or those with a family history of PD, enabling earlier intervention and better management of the disease. The growing evidence for α-synuclein as a biomarker opens the door to a future where PD can be diagnosed and treated earlier, improving patients’ quality of life and potentially altering the course of the disease for many. New studies should continue to be assessed in the future as more and more research is conducted on this topic and further advancements are made in diagnosing PD.

## Figures and Tables

**Table 1:** Summary of the above studies on α-synuclein amplification assays for Parkinson’s disease.

Author/Year	Study Objective	Results
University of Edinburg—2016 [39]	Develop an assay that detects aggregations of α-synuclein in the CSF and brain tissue from patients diagnosed with dementia with Lewy bodies (DLB) or PD.	The assay was found to have 95% sensitivity in detecting PD. Assays were negative for dysfunctional tau rather than α-synuclein disorders. The first study demonstrates the possible clinical utility of a diagnostic test for PD.
University of Texas—2020 [40]	Determine if the assay can differentiate between the CSF of someone with PD & someone with MSA.	Aggregation of α-synuclein was significantly higher in those with PD than those with MSA.
Amprion—2021 [41,42]	Longitudinal collection of biological samples from cohorts with different PD status to determine the sensitivity and specificity of the assay.	The assay was found to have 96.2% sensitivity and 96.7% specificity for PD versus control at baseline and 96.4% sensitivity and 93.8% specificity three years later. α-synuclein PMCA assay is very accurate in determining PD from individuals with normal DaTscans and may be able to detect changes before they may be seen on a scan, enabling an earlier diagnosis and, therefore, treatment
Amprion, AbbVie, Caughey Comparison [36]	Determine the reproducibility of the assay across providers and labs.	Reference Table 1.
Qatar Biomedical Research Institute—2022 [43]	Efficacy of combining α-synuclein SAA with ELISA to provide a quantitative test result that correlates with disease severity.	Strong evidence was found that adding ELISA to SAA may be utilized to make an earlier diagnosis and quantify disease severity.

**Table 2:** Recent studies exploring α-synuclein Amplification Assays for Parkinson’s disease.

Author/Year	Study Objective	Groups Studied	Results and Findings
(Kuzkina et al., 2021) [44]	To determine the reliability and reproducibility of skin biopsies in RT-QuIC for diagnosis of PD	34 PD patients, 30 controls	Diagnostic accuracy for PD: 88.9% Agreement between two laboratories: 92.2% Results of the assay correlated with disease progression
(Rossi et al., 2020) [45]	To determine the reliability of CSF in RT-QuIC for the diagnosis of synucleinopathies across a large cohort	439 CSF samples total, consisting of DLB, AD, PSP, MSA, PD, RBD, and PAF patients	Diagnostic accuracy for synucleinopathies: 95.3%
(Orrù et al., 2021) [46]	To test the BioFIND assay (an improved RT-QuIC) in the diagnosis of PD	108 PD patients, 85 controls	Assay gave a positive result in 97% of PD patients and 13% of controls Results weakly correlated with disease progression. Improved assay reduces reaction time from 5–13 days down to 1–2 days

RT-QuIC: real-time quaking-induced conversion; PD: Parkinson’s disease; CSF: cerebrospinal fluid; DLB: dementia with Lewy bodies; AD: Alzheimer’s disease; PSP: progressive supranuclear palsy; MSA: multiple system atrophy; RBD: REM sleep behavior disorder; PAF: pure autonomic failure.

## References

[1] Hayes M.T. (2019). Parkinson’s Disease and Parkinsonism. Am. J. Med..

[2] Jankovic J. (2008). Parkinson’s disease: Clinical features and diagnosis. J. Neurol. Neurosurg. Psychiatry.

[3] Kouli A., Torsney K.M., Kuan W.L., Stoker T.B., Greenland J.C. (2018). Parkinson’s Disease: Etiology, Neuropathology, and Pathogenesis. Parkinson’s Disease: Pathogenesis and Clinical Aspects.

[4] Willis A.W., Roberts E., Beck J.C., Fiske B., Ross W., Savica R., Eeden S.K.V.D., Tanner C.M., Marras C., Alcalay R. (2022). Incidence of Parkinson disease in North America. Npj Park. Dis..

[5] Yang W., Hamilton J.L., Kopil C., Beck J.C., Tanner C.M., Albin R.L., Dorsey E.R., Dahodwala N., Cintina I., Hogan P. (2020). Current and Projected Future Economic Burden of Parkinson’s Disease in the U.S. Npj Park. Dis..

[6] Gomes B.F., Farris C.M., Ma Y., Concha-Marambio L., Lebovitz R., Nellgård B., Dalla K., Constantinescu J., Constantinescu R., Gobom J. (2023). α-Synuclein seed amplification assay as a diagnostic tool for parkinsonian disorders. Park. Relat. Disord..

[7] Coelho M., Ferreira J.J. (2012). Late-stage Parkinson disease. Nat. Rev. Neurol..

[8] Goedert M. (2001). Alpha-synuclein and neurodegenerative diseases. Nat. Rev. Neurosci..

[9] Noyce A.J., Bestwick J.P., Silveira-Moriyama L., Hawkes C.H., Giovannoni G., Lees A.J., Schrag A. (2012). Meta-analysis of early nonmotor features and risk factors for Parkinson disease. Ann. Neurol..

[10] Islam S., Azim F., Saju H., Zargaran A., Shirzad M., Kamal M., Fatema K., Rehman S., Azad M.M., Ebrahimi-Barough S. (2021). Pesticides and Parkinson’s disease: Current and future perspective. J. Chem. Neuroanat..

[11] Gasser T. (2009). Molecular pathogenesis of Parkinson disease: Insights from genetic studies. Expert Rev. Mol. Med..

[12] Xu W., Tan L., Yu J.-T. (2015). The link between the SNCA gene and parkinsonism. Neurobiol. Aging.

[13] Benson D.L., Matikainen-Ankney B.A., Hussein A., Huntley G.W. (2018). Functional and behavioral consequences of Parkinson’s disease-associated *LRRK2*-G2019S mutation. Biochem. Soc. Trans..

[14] Zhu W., Huang X., Yoon E., Bandres-Ciga S., Blauwendraat C., Billingsley K.J., Cade J.H., Wu B.P., Williams V.H., Schindler A.B. (2022). Heterozygous *PRKN* mutations are common but do not increase the risk of Parkinson’s disease. Brain.

[15] Dolgacheva L.P., Berezhnov A.V., Fedotova E.I., Zinchenko V.P., Abramov A.Y. (2019). Role of DJ-1 in the mechanism of pathogenesis of Parkinson’s disease. J. Bioenerg. Biomembr..

[16] Diagnosis and the Premotor Phase of Parkinson’s Disease|Neurology. https://www.neurology.org/doi/abs/10.1212/wnl.0b013e318198db11?casa_token=eVV5bkh9VlAAAAAA%3APPxUrx9ENhu3u4-8zJ3jyYWPslnkYbnawirEOk92GqUVJyBuwAItpAGsbm7rYlw86A0pkrrNViE4b88.

[17] Armstrong M.J., Okun M.S. (2020). Diagnosis and Treatment of Parkinson Disease. JAMA.

[18] Fearnley J.M., Lees A.J. (1991). Ageing and parkinson’s disease: Substantia nigra regional selectivity. Brain.

[19] Tysnes O.-B., Storstein A. (2017). Epidemiology of Parkinson’s disease. J. Neural Transm..

[20] Parnetti L., Gaetani L., Eusebi P., Paciotti S., Hansson O., El-Agnaf O., Mollenhauer B., Blennow K., Calabresi P. (2019). CSF and blood biomarkers for Parkinson’s disease. Lancet Neurol..

[21] Adler C.H., Beach T.G., Hentz J.G., Shill H.A., Caviness J.N., Driver-Dunckley E., Sabbagh M.N., Sue L.I., Jacobson S.A., Belden C.M. (2014). Low clinical diagnostic accuracy of early vs advanced Parkinson disease. Neurology.

[22] Kang U.J., Boehme A.K., Fairfoul G., Shahnawaz M., Ma T.C., Hutten S.J., Green A., Soto C. (2019). Comparative study of cerebrospinal fluid α-synuclein seeding aggregation assays for diagnosis of Parkinson’s disease. Mov. Disord..

[23] Kluge A., Bunk J., Schaeffer E., Drobny A., Xiang W., Knacke H., Bub S., Lückstädt W., Arnold P., Lucius R. (2022). Detection of neuron-derived pathological α-synuclein in blood. Brain.

[24] Srinivasan E., Chandrasekhar G., Chandrasekar P., Anbarasu K., Vickram A.S., Karunakaran R., Rajasekaran R., Srikumar P.S. (2021). Alpha-Synuclein Aggregation in Parkinson’s Disease. Front. Med..

[25] Chang K.-H., Chen C.-M. (2020). The Role of Oxidative Stress in Parkinson’s Disease. Antioxidants.

[26] Holdorff B. (2002). Friedrich Heinrich Lewy (1885–1950) and His Work. J. Hist. Neurosci..

[27] Uversky V.N. (2007). Neuropathology, biochemistry, and biophysics of α-synuclein aggregation. J. Neurochem..

[28] Goldberg M.S., Lansbury P.T. (2000). Is There a cause-and-effect relationship between α-synuclein fibrillization and parkinson’s disease?. Nat. Cell Biol..

[29] Wood S.J., Wypych J., Steavenson S., Louis J.-C., Citron M., Biere A.L. (1999). α-Synuclein Fibrillogenesis Is Nucleation-dependent: Implications for the pathogenesis of parkinson′s disease. J. Biol. Chem..

[30] Power J.H.T., Barnes O.L., Chegini F. (2017). Lewy Bodies and the Mechanisms of Neuronal Cell Death inParkinson’s Disease and Dementia withLewy Bodies. Brain Pathol..

[31] Kalia L.V. (2019). Diagnostic biomarkers for Parkinson’s disease: Focus on α-synuclein in cerebrospinal fluid. Park. Relat. Disord..

[32] Bellomo G., De Luca C.M.G., Paoletti F.P., Gaetani L., Moda F., Parnetti L. (2022). α-Synuclein Seed Amplification Assays for Diagnosing Synucleinopathies. Neurology.

[33] Shi M., Liu C., Cook T.J., Bullock K.M., Zhao Y., Ginghina C., Li Y., Aro P., Dator R., He C. (2014). Plasma exosomal α-synuclein is likely CNS-derived and increased in Parkinson’s disease. Acta Neuropathol..

[34] Höglund K., Fourier A., Perret-Liaudet A., Zetterberg H., Blennow K., Portelius E. (2015). Alzheimer’s disease—Recent biomarker developments in relation to updated diagnostic criteria. Clin. Chim. Acta.

[35] Galasko D.R., Shaw L.M. (2017). CSF biomarkers for Alzheimer disease—Approaching consensus. Nat. Rev. Neurol..

[36] Russo M.J., Orru C.D., Concha-Marambio L., Giaisi S., Groveman B.R., Farris C.M., Holguin B., Hughson A.G., LaFontant D.-E., Caspell-Garcia C. (2021). High diagnostic performance of independent alpha-synuclein seed amplification assays for detection of early Parkinson’s disease. Acta Neuropathol. Commun..

[37] Siderowf A., Concha-Marambio L., Lafontant D.-E., Farris C.M., Ma Y., A Urenia P., Nguyen H., Alcalay R.N., Chahine L.M., Foroud T. (2023). Assessment of heterogeneity among participants in the Parkinson’s Progression Markers Initiative cohort using α-synuclein seed amplification: A cross-sectional study. Lancet Neurol..

[38] Siderowf A. α-Synuclein Seed Amplification in a Large Parkinson Cohort. https://www.neurology.org/media/podcast/_-synuclein-seed-amplification-large-parkinson-cohort.

[39] Fairfoul G., McGuire L.I., Pal S., Ironside J.W., Neumann J., Christie S., Joachim C., Esiri M., Evetts S.G., Rolinski M. (2016). Alpha-synuclein RT-QuIC in the CSF of patients with alpha-synucleinopathies. Ann. Clin. Transl. Neurol..

[40] Shahnawaz M., Mukherjee A., Pritzkow S., Mendez N., Rabadia P., Liu X., Hu B., Schmeichel A., Singer W., Wu G. (2020). Discriminating α-synuclein strains in Parkinson’s disease and multiple system atrophy. Nature.

[41] About|Parkinson’s Progression Markers Initiative. https://www.ppmi-info.org/about-ppmi.

[42] Concha-Marambio L., Farris C.M., Holguin B., Ma Y., Seibyl J., Russo M.J., Kang U.J., Hutten S.J., Merchant K., Shahnawaz M. (2021). Seed Amplification Assay to Diagnose Early Parkinson’s and Predict Dopaminergic Deficit Progression. Mov. Disord..

[43] Majbour N., Aasly J., Abdi I., Ghanem S., Erskine D., van de Berg W., El-Agnaf O. (2022). Disease-Associated a-Synuclein Aggregates as Biomarkers of Parkinson Disease Clinical Stage. Neurology.

[44] Kuzkina A., Bargar C., Schmitt D., Rößle J., Wang W., Schubert A.-L., Tatsuoka C., Gunzler S.A., Zou W.-Q., Volkmann J. (2021). Diagnostic value of skin RT-QuIC in Parkinson’s disease: A two-laboratory study. Npj Park. Dis..

[45] Rossi M., Candelise N., Baiardi S., Capellari S., Giannini G., Orrù C.D., Antelmi E., Mammana A., Hughson A.G., Calandra-Buonaura G. (2020). Ultrasensitive RT-QuIC assay with high sensitivity and specificity for Lewy body-associated synucleinopathies. Acta Neuropathol..

[46] Orrù C.D., Ma T.C., Hughson A.G., Groveman B.R., Srivastava A., Galasko D., Angers R., Downey P., Crawford K., Hutten S.J. (2020). A rapid α-synuclein seed assay of Parkinson’s disease CSF panel shows high diagnostic accuracy. Ann. Clin. Transl. Neurol..

